# Novel piperazine‐based ionizable lipid nanoparticles allow the repeated dose of mRNA to fibrotic lungs with improved potency and safety

**DOI:** 10.1002/btm2.10556

**Published:** 2023-05-28

**Authors:** Minjeong Kim, Michaela Jeong, Gyeongseok Lee, Yeji Lee, Jeongeun Park, Hyein Jung, Seongeun Im, Joo‐Sung Yang, Kyungjin Kim, Hyukjin Lee

**Affiliations:** ^1^ College of Pharmacy, Graduate School of Pharmaceutical Sciences Ewha Womans University Seoul South Korea; ^2^ Department of Biochemistry, Simmons Comprehensive Cancer Center The University of Texas Southwestern Medical Center Dallas Texas USA; ^3^ ST Pharm Co. Ltd. Seoul South Korea

**Keywords:** gene expression, ionizable lipids, lipid nanoparticles, mRNA, pulmonary fibrosis

## Abstract

mRNA‐based protein replacement therapy has received much attention as a novel intervention in clinical disease treatment. Lipid nanoparticles (LNPs) are widely used for their therapeutic potential to efficiently deliver mRNA. However, clinical translation has been hampered by the immunogenicity of LNPs that may aggravate underlying disease states. Here, we report a novel ionizable LNP with enhanced potency and safety. The piperazine‐based biodegradable ionizable lipid (244cis) was developed for LNP formulation and its level of protein expression and immunogenicity in the target tissue was evaluated. It was found that 244cis LNP enabled substantial expression of the target protein (human erythropoietin), while it minimally induced the secretion of monocyte chemoattractant protein 1 (MCP‐1) as compared to other conventional LNPs. Selective lung targeting of 244cis LNP was further investigated in tdTomato transgenic mice with bleomycin‐induced pulmonary fibrosis (PF). The repeated administration of 244cis LNP with Cre recombinase mRNA achieved complete transfection of lung endothelial cells (~80%) and over 40% transfection of Sca‐1‐positive fibroblasts. It was shown that 244cis LNP allows the repeated dose of mRNA without the loss of activity due to its low immunogenicity. Our results demonstrate that 244cis LNP has great potential for the treatment of chronic diseases in the lungs with improved potency and safety.

## 1 INTRODUCTION

In vitro transcript (IVT) mRNA is widely utilized as a powerful tool to express a target protein in cells for therapeutic and biomedical application.^1^, ^2^ However, clinical translation of mRNA has been hindered by the difficulty of its delivery due to its large size, negative charge, and chemical instability.^2^ To broaden its applications, efficient delivery systems are necessary to facilitate the intracellular delivery of mRNA to the target cells, while maintaining its stability during and after systemic administration.^3^ In this regard, lipid nanoparticles (LNPs) show great promise and they are widely used for the delivery of various RNA therapeutics.^3^, ^4^

Several ionizable lipids have been developed and applied in therapeutic treatments. For example, DLin‐MC3‐DMA (MC3) has been utilized in Onpattro, the first United States Food and Drug Administration‐approved RNAi therapeutic.^5^, ^6^ Successful hTTR gene silencing was achieved for patients who received Onpattro intravenously every 3 weeks over 18 months.^6^, ^7^ More recently, various ionizable lipids, including MC3 and SM‐102, were investigated for in vivo mRNA delivery.^8^ These studies revealed that MC3 is not sufficient for high expression of target proteins. Contrarily, SM‐102 and its derivatives showed substantial protein expression in target tissues as compared to that of MC3.^8^ According to the study, the balance between the potency of LNP and its immunogenicity is the most important factor for the consideration of repeated RNA doses and postinjection safety.

In the treatment of chronic diseases, the immunogenicity of LNP can aggravate the underlying disease state and may reduce the effects of mRNA therapeutics. Therefore, it is challenging for LNP to enable the repeated administration of mRNA with high potency and safety. In this study, we report a novel biodegradable ionizable lipid (244cis) for the formulation of an LNP that allows substantial expression of target proteins with minimal immune activation. It was found that the 244cis LNP showed superior protein expression as compared with two conventional LNPs (MC3 and SM‐102). The characteristics of each LNP are well explained by the plot consisting of human erythropoietin (hEPO) expression versus cytokine release.

**FIGURE 1:**
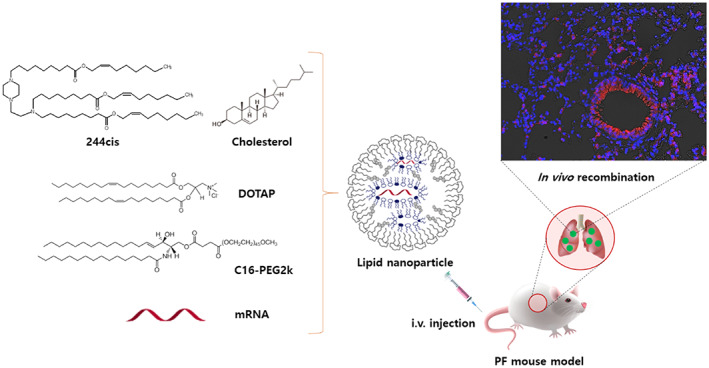
The illustration of newly developed ionizable lipid nanoparticles (LNPs) for targeted delivery of mRNA to the lungs of idiopathic pulmonary fibrosis (IPF) mice. The new biodegradable LNP can deliver mRNA safely and efficiently to the pulmonary fibrosis‐induced lungs of mice by systemic administration.

A highly aggressive pulmonary fibrosis (PF) animal model was utilized to investigate how disease‐compromised lung physiology can affect the delivery of mRNA by the selective organ targeting LNPs. 244cis was used for the formulation of lung targeting LNPs with additional cationic helper lipids. The optimized formulation for lung delivery was verified by achieving over 80% lung specific expression of firefly luciferase (fLuc). The induction of cytokines, monocyte chemoattractant protein 1 (MCP‐1), was monitored, and its level was much lower than that of SM‐102. It was demonstrated that the repeated dose of 244cis LNP with Cre recombinase mRNA showed nearly complete transfection of endothelial cells in the lungs of tdTomato transgenic mice. This phenomenon was consistently observed in cases of both nonfibrosis and fibrosis models. A substantial level of tdTomato expression was achieved in lung endothelial cells, fibroblasts, epithelial cells, and immune cells. It was also noticeable that mRNA delivery to the Sca‐1‐positive fibroblasts occurred in the fibrosis model. Overall, we demonstrated that 244cis LNP can be a superior candidate for repeated mRNA doses and may offer sustainable protein replacement therapy to treat chronic diseases of the lungs, such as idiopathic pulmonary fibrosis (Figure 1).

## 2 RESULTS AND DISCUSSION

In this study, a novel biodegradable ionizable lipid (244cis) was synthesized and used for the preparation of the LNP formulation. First, the (Z)‐non‐2‐en‐1‐yl 9‐bromononanoate tail was synthesized by an esterification reaction with 9‐bromononanoic acid and cis‐2‐nonen‐1‐ol. A piperazine derivative (244) was reacted with the brominated carbon tail to obtain the 244cis ionizable lipid (244cis) via an S_N_2 reaction. The synthesis scheme is given in Figure S1. Also, the lipid structure was characterized by NMR (Figure S2). The 244cis ionizable lipid has unique structural properties characterized by a piperazine‐based amine head and unsaturated lipid tails. We assumed these properties could affect the endosomal release of LNPs by enhancing membrane fluidity. In particular, unsaturated lipids are known to enhance membrane fluidity upon fusion with the endosomal membrane, potentially facilitating the release of LNPs.^9^ Therefore, we performed a fluorescence resonance energy transfer (FRET) assay at pH 5.5 to determine the lipid fusion ability of 244cis LNP and SM‐102 LNP. Our results showed that the 244cis LNP outperformed the SM‐102 LNP by exhibiting up to 30% of lipid fusion. This indicates that 244cis LNP can provide facilitated disassembly of endosomal membrane for mRNA delivery into the cytosol (Figure S3).^10^

For the optimization of LNP formulation, we compared the potency of 244cis LNP with two different PEG lipids: C16‐PEG2k ceramide and DMG‐PEG2k. Our previous study revealed that the C16‐PEG2k ceramide was the most suitable choice for the formulation of ionizable lipids with the piperazine structure.^11^ When directly compared to the 244cis LNP with DMG‐PEG2k, we found that the LNP with C16‐PEG2K ceramide show slightly higher potency with no significant difference between the tested groups (Figure S4).

To evaluate the superior potency of 244cis over conventional ionizable lipids (MC3 and SM‐102), hEPO encoding mRNA was formulated with different types of LNPs and intravenously administered at a dose of 0.1 mg/kg. After 6 h, blood was collected and the hEPO and MCP‐1 serum levels were validated. hEPO is a secretory protein and evaluating its concentration in serum allows the quantitative analysis of LNP delivery potency.^8^ On the other hand, MCP‐1 is an important chemokine for the initiation of inflammation.^12^ Thus, the upregulation of serum MCP‐1 indicates acute inflammatory responses following LNP administration.

**FIGURE 2:**
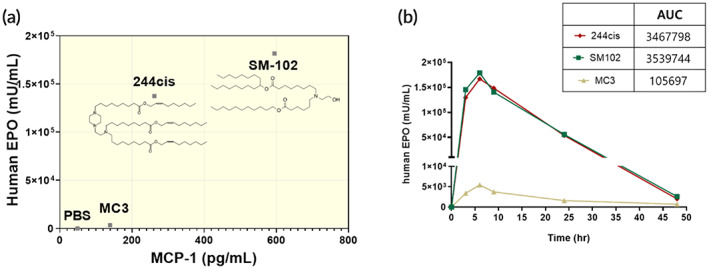
The 244cis lipid nanoparticles (LNPs) show improved potency of mRNA delivery and translation without immunogenicity. (a) The graph represents the potency versus immunogenicity of three different LNPs (244cis, SM‐102, and MC3). On the *y*‐axis, serum expression level of human erythropoietin (hEPO) protein is plotted against the serum level of monocyte chemoattractant protein 1 (MCP‐1) levels on the *x*‐axis. Three LNPs were systemically injected at the dose of 0.1 mg/kg of hEPO mRNA. After 6 h injection, the serum level of hEPO and MCP‐1 was analyzed by ELISA (*n* = 5, mean ± standard deviation [*SD*]). (b) The expression profile of hEPO by three LNPs at different time points. Mice were injected with LNPs containing 0.1 mg/kg of hEPO mRNA via systemic administration. SM‐102 and MC3 were used as controls (*n* = 4, mean ± *SD*).

As shown in Figure 2a, the 244cis LNP expressed 1.3 × 10^5^ mU/mL of hEPO protein. It induced 25‐fold higher protein expression than the MC3 and was comparable to the SM‐102 LNPs. More importantly, the 244cis LNP showed a marginal level of MCP‐1 secretion (262 pg/mL) as compared to SM‐102 (594 pg/mL). This potency versus immunogenicity plot clearly shows the characteristics of each LNP and may indicate the appropriate uses of LNPs for different applications, such as vaccine and protein replacement therapy.^13^ The expression profile of hEPO by the 244cis LNP was further analyzed for 2 days and compared with that of SM‐102 and MC3 (Figure 2b). The expression of hEPO in serum peaked at 6 h and decreased slowly over 48 h. Similar expression trends were observed for the MC3 and SM‐102 LNPs. Again, a noticeable increase in hEPO expression was observed with the 244cis LNP (AUC: 3.4^6^ mU/mL) relative to the SM‐102 and MC3 LNPs (AUC: 3.5^6^, 1.0^5^ mU/mL, respectively). To elicit the desired therapeutic response, mRNA mediated target protein expression requires a minimum plasma concentration, and the concentration of protein needs to be maintained within the therapeutic window.^14^ Previously, Sabnis et al. have demonstrated the possibility of multiple mRNA doses with SM‐102.^8^ However, previous studies reported that the activation of the immune system markedly limits the repeated dose of mRNA‐loaded LNP.^15^ In our study, we demonstrated that the expression profile of hEPO in serum was consistent after the repeated injections of hEPO mRNA (0.1 mg/kg) at 2‐week intervals. Upon the repeated dose of mRNA, the hEPO expression in serum was well maintained by the 244cis LNP in ICR mice (Figure S5). Considering that the 244cis LNP showed a similar expression profile to that of SM‐102 with lower immunogenicity, we conclude that the 244cis LNP is a superior candidate for the repeated dose of mRNA.

**FIGURE 3:**
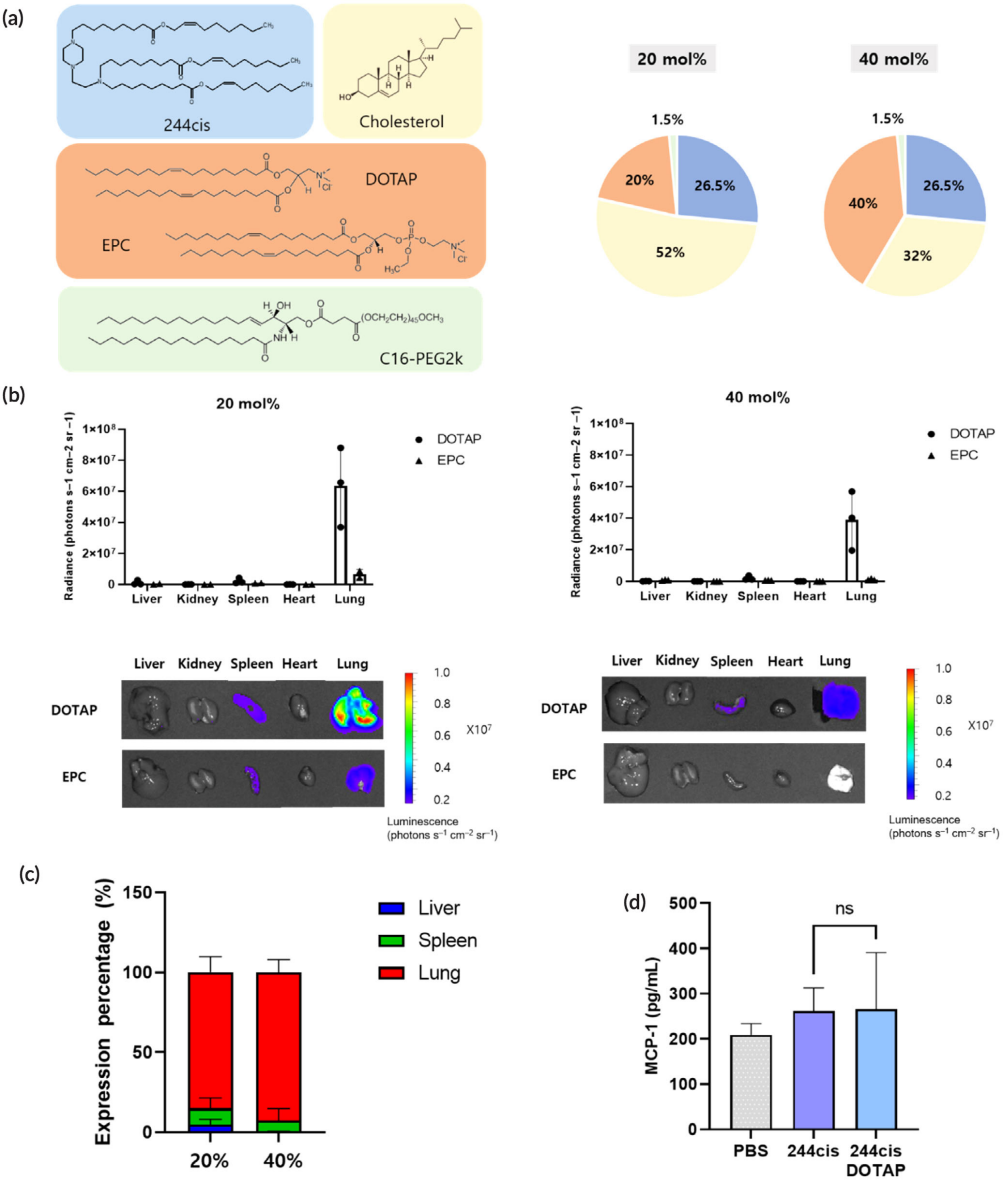
Optimization of lung targeting LNPs with the replacement of helper lipid (a) Chemical structures of four lipid components and the molar ratio of the lung targeting LNPs. LNPs were formulated with 20 or 40 mol% of cationic lipids instead of DOPE. (b) Evaluation of delivery efficacy to the lungs by firefly luciferase expression. The LNPs were loaded with fLuc mRNA and injected systemically into C57BL/6 mice at the mRNA dose of 0.2 mg/kg. After 6 h of injection, bioluminescence was analyzed by ex vivo organ imaging (*n* = 3, mean ± SD). (c) The lung targeting LNPs containing 20% of DOTAP accumulated in the lungs selectively. (d) Evaluation of serum MCP‐1 expression upon the administration of lung targeting LNPs as compared to their native LNPs. The LNPs were loaded with hEPO mRNA and injected systemically into BALB/c mice at an mRNA dose of 0.1 mg/kg. After 6 h of injection, the blood was collected, and MCP‐1 cytokines were analyzed by ELISA (*n* = 3–4, mean ± *SD*).

Previous studies have demonstrated that the addition of positively charged helper lipids to the LNP formulation can alter passive organ targeting from the liver to the lungs.^16^, ^17^ For the conventional LNP, ApoE protein adsorbs on the surface of LNP and interacts with the LDL receptor overexpressed hepatocytes for cellular uptake. However, incorporating cationic lipid with an ammonium headgroup can alter the surface characteristics of LNP and affects the profile of plasma protein adsorption on the surface of LNP. As a result, negatively charged proteins in plasma with low isoelectric point, such as Vtn or Pon1, are adsorbed on the surface of LNP and enable the targeted delivery of LNP to the lung.^18^ Consequently, the formulation of the 244cis LNP was optimized to ensure the successful delivery of mRNA to the lungs. Two different cationic helper lipids (1,2‐dioleoyl‐3‐trimethylammonium propane [DOTAP] and ethyl phosphocholine [EPC]) were selected instead of 1,2‐dioleoyl‐sn‐glycero‐3‐phosphoethanolamine [DOPE] to formulate the lung targeting LNPs (Figure 3a). Two different molar ratios of the cationic helper (20% and 40%) were investigated to validate their effects on the selective lung targeting of LNP. As shown in Figure S6, all prepared LNPs had an encapsulation efficiency of >90% and a diameter of <100 nm. As previously reported, the addition of cationic helper lipids did not affect the size of the LNP, but a slight change in the surface charge was observed.^17^

When tested in vivo using firefly luciferase encoding mRNA (fLuc), the 244cis LNP containing 20% DOTAP showed substantial fLuc expression in the lungs (Figure 3b). Selective organ targeting was verified by evaluating the fLuc expression in three different organs, including the liver, spleen, and lungs. Over 80% fLuc expression was observed in the lungs by incorporating 20% and 40% of DOTAP (Figure 3c). When we tested the formulation of SM‐102 LNP with DOTAP, it was found that the targeting efficiency was around 60% fluc expression in the lung, which is less than 244cis LNP (Figures S7 and S8). Next, the immunogenicity of the lung targeting LNPs was further examined by testing MCP‐1 levels in serum. Blood was collected after 6 h of intravenous administration (0.1 mg/kg). The lung targeting LNPs, which was incorporated with DOTAP, resulted in a marginal increase of serum MCP‐1 levels (272.6 pg/mL), comparable to that of the 244cis DOPE LNP (262.1 pg/mL) (Figure 3d). In addition, histological analysis has confirmed the safety of 244cis DOTAP LNP in the lung (Figure S9). No significant pathological differences were observed after the administration of 244cis DOTAP LNP including immune cell recruitment and inflammation.

**FIGURE 4:**
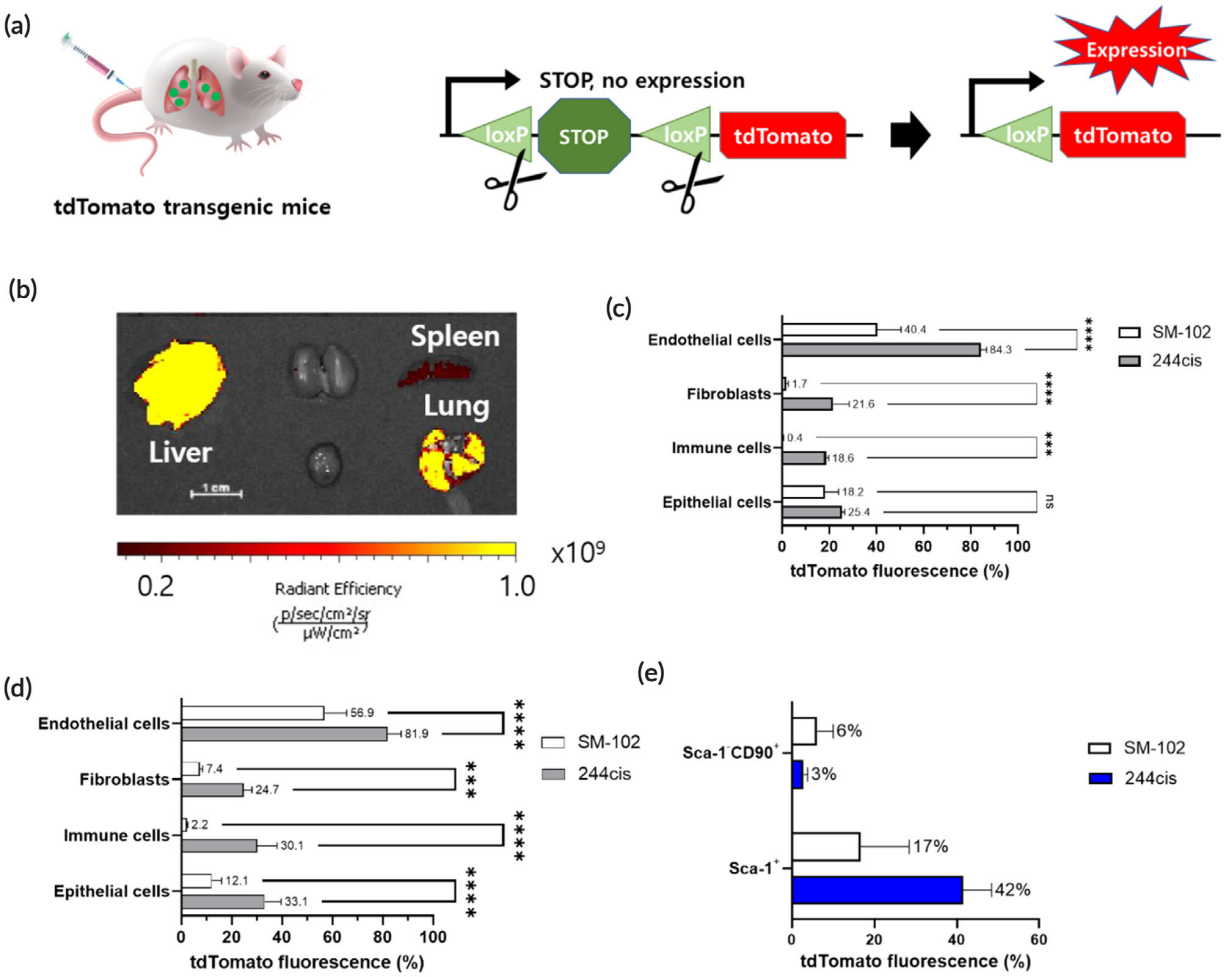
Evaluation of cell type‐specific delivery of Cre mRNA in the lungs. (a) The schematic illustration shows that the deletion of the stop cassette by the delivery of Cre mRNA activating the expression of tdTomato. (b) The lung targeting LNPs carrying 0.3 mg/kg Cre mRNA were systemically administrated, resulting in the expression of tdTomato fluorescence in the lungs. Mice were sacrificed, and five major organs were imaged for tdTomato fluorescence 72 h after IV injection. (c) Flow cytometry analysis of cell type‐specific delivery of Cre mRNA using 244cis or SM‐102 LNPs in nonpulmonary fibrosis (PF) and (d) PF models to quantify the percentage of tdTom+ cells (*n* = 3, mean ± *SD*, ****p < 0.0001, one‐way analysis of variance [ANOVA]). (e) Flow cytometry analysis of tdTom+ cells in the subtypes of fibroblasts (*n* = 3, mean ± *SD*).

To further investigate the lung‐targeted delivery of mRNA under chronic disease conditions, such as pulmonary fibrosis, tdTomato transgenic mice were evaluated under nonfibrosis and fibrosis conditions. Cre mRNA‐loaded LNP was administered to the mice at the mRNA dose of 0.3 mg/kg. After 2 days, tdTomato fluorescence was analyzed by in vivo imaging. To confirm the potential of repeated dosing, 244cis LNPs were administered twice at 4‐day intervals (Figure S10a). Once Cre recombinase is expressed in the target cells, it excises LoxP on either side of the stop codon and turns on the expression of the tdTomato protein (Figure 4a).^19^ Therefore, expression of tdTomato allows facile analysis of cells transfected with LNP. As shown in Figure 4b, robust tdTomato fluorescence was detected in the lungs. Although some level of fluorescence was detected in the liver, it was negligible compared to that of the DOPE LNP (Figure S11).

Next, the cell type‐specific Cre‐mediated recombination was evaluated within the lungs, and single cells were isolated and analyzed using flow cytometry (Figure 4c). Under the nonfibrosis condition, the 244cis LNP transfected ~84% of endothelial cells, ~21% of fibroblasts, ~18% of immune cells, and ~25% of epithelial cells in the lungs, while the SM‐102 LNP showed slightly low recombination occurrence in the lungs (~40% of endothelial cells, ~1.7% of fibroblasts, ~0.4% of immune cells, and ~18.2% of epithelial cells). The potency of 244cis LNP was substantial higher as compared that of over SM‐102 LNP, and there were significant differences in all tested types of lung cells except epithelial cells. To induce pulmonary fibrosis, bleomycin was intratracheally injected into the mice at a dose of 1.8 mg/kg. As shown in Figure S12, bleomycin induced deposition of interstitial collagen in the lungs. After 10 days, Cre mRNA‐loaded LNPs were injected twice at 4‐day intervals (Figure S10b). Cell type‐specific delivery potency of LNP was again quantified by flow cytometry. As shown in Figure 4d, the 244cis LNP resulted in 82% of endothelial cells, 25% of fibroblasts, 30% of immune cells, and 33% of epithelial cells becoming tdTomato‐positive. The data corresponded well with the in vivo recombination profile in the lungs of the nonfibrosis model. It was noticeable that the 244cis LNP showed statistically higher in vivo transfection potency compared to that of SM‐102 LNP. This observation was consistent in the case of wild type and fibrosis model mouse.

Lastly, the interaction between the 244cis LNP with specific lung fibroblasts was investigated. It was found that the 244cis LNP transfected 42% of Sca‐1‐positive fibroblasts and 3% of Sca‐1‐negative and CD90‐positive fibroblasts (Figure 4e). It is noticeable that 244cis LNP showed much higher transfection in Sca‐1‐positive fibroblasts as compared to the SM‐102 LNP. Previous studies revealed that Sca‐1 positive fibroblasts with CD248 fibroblasts are predominantly distributed in the collagen fiber‐rich connective tissue of fibroblastic foci and play an essential role in the progression of fibrosis by proliferating fibroblasts.^20^, ^21^ Since these studies demonstrated that the down regulation of CD248 expression on the fibroblast cells could reduce cell proliferation, the Sca‐1‐positive fibroblasts can be a target cells for RNA delivery in the lungs. Therefore, 244cis LNP may offer targeted delivery of RNA to the Sca‐1 positive fibroblasts and regulate the fibrosis progression under disease condition.

## 3 CONCLUSION

The targeted delivery of mRNA to the lungs is an attractive strategy for treating pulmonary diseases through protein replacement and gene editing.^1^, ^22^ However, there is an urgent need for the development of an LNP that readily delivers therapeutic mRNA to different types of pulmonary cells. To improve the delivery of mRNA to the fibrotic lungs, we aimed to develop a novel LNP that is well acceptable in the treatment of chronic diseases. The acute immune response following LNP administration may exacerbate the underlying disease state of PF and decrease the delivery potency of the LNP. Therefore, it is important to achieve superior potency of protein expression with minimal immunogenicity to enable the repeated dose of mRNA.^8^, ^23^

In this study, we demonstrated efficient mRNA delivery to the lungs of PF model mice using the 244cis LNP. During fibrosis progression, immune cell infiltration and formation of fibrotic tissue are detected.^24^ This may affect the interactions between the pulmonary cells and intravenously delivered LNP. However, the 244cis LNP offers substantial target protein expression in pulmonary cells, such as endothelial cells, fibroblasts, epithelial cells, and immune cells. To our knowledge, there have been no reports of an LNP that can efficiently deliver mRNA to the lungs of PF model mice with high potency. It is likely that the low immunogenicity of the 244cis LNP plays an important role. Thus, it allows a repeated mRNA dose without the loss of high potency. More work remains to be investigated on how the structure of ionizable lipids causes LNP immunogenicity. Finally, our study shows that the 244cis LNP has enormous potential for the repeated delivery of mRNA to treat chronic diseases of the lungs.

## 4 METHODS

### 4.1 Materials

Reporter mRNAs (fLuc mRNA, Cre mRNA, and hEPO mRNA) used in this study were purchased from TriLink BioTechnologies (L‐7602, L‐7211, L‐7209; San Diego, CA, USA). hEPO enzyme‐linked immunosorbent assay (ELISA) kits were purchased from R&D Systems (DEP00; Minneapolis, MN, USA). MCP‐1 ELISA kits were purchased from Thermo Fisher (BMS281; Waltham, MA, USA). Liberase TM Research Grade (5401127001), DNase I (4716728001), and hyaluronidase (H3506) were purchased from Sigma‐Aldrich (St. Louis, MO, USA). Bleomycin Sulfate (1076308) was purchased from USP (Rockville, MD, USA).

### 4.2 Preparation of mRNA‐loaded LNPs

Synthesis of 244cis is described in Figure S1. mRNA‐loaded LNPs were formulated via a microfluidic device as previously reported.^11^ Briefly, ionizable lipids, helper lipids, cholesterol, and PEG‐lipids were diluted in ethanol. The molar ratio between lipid components was determined according to previously reported studies. To prepare lung targeting LNPs, helper lipids were replaced with cationic helper lipids (EPC or DOTAP). Taking DOTAP containing LNPs as an example, the weight ratio of ionizable lipids to mRNA was 20:1, and the molar ratio between ionizable lipids:DOTAP:Cholesterol:PEG‐lipid was 26.5:20:52:1.5 or 26.5:40:32:1.5. mRNA was diluted in citrate buffer (10 mM, pH 3). The volume ratio between ethanol and citrate buffer was 1:3. Two solutions were then mixed via a microfluidic device at a 12 mL/min ratio. After LNP formation, the resultant LNPs were diluted in a 40‐fold volume of 1× phosphate‐buffered saline and concentrated via ultrafiltration. mRNA‐loaded LNPs were further characterized for physical properties (mRNA encapsulation efficiency, size, and polydispersity index [PDI]).

### 4.3 Lipid fusion ability by FRET assay

A FRET assay was used with endosomal mimicking anionic liposomes. The anionic liposomes were prepared by combining DOPS, DOPC, DOPE, NBD‐PE, and Rho‐PE in a 25:25:48:1:1 molar ratio, as followed by the previous study.^10^ Both 244cis LNP and SM‐102 LNP were formulated with ionizable lipid concentration of 1mM. Then PBS with pH 5.5 was added to black 96‐well plates at a volume of 100 μL per well. One microliter of endosomal mimicking anionic liposomes was added to each well, followed by 10 μL of LNPs. The mixture was incubated at 37°C for 5 min, and fluorescence measurements were taken using a microplate reader (GloMax®; Promega, Madison, WI, USA) at Ex/Em = 465/520 nm.

### 4.4 In vivo mRNA delivery

Seven‐week‐old female C57BL/6 mice were purchased from Orient Bio (Seongnam, South Korea). Mice were administered fLuc mRNA‐loaded LNPs via retro‐orbital injection at an mRNA dose of 0.2 mg/kg. Six hours later, mice were injected intraperitoneally with 200 μL of 30 mg/mL d‐luciferin (VivoGlo™ Luciferin; Promega, Madison, WI, USA). Twenty minutes later, mice were euthanized by CO_2_ inhalation. Five major organs (heart, lungs, liver, spleen, and kidney) were collected, and luminescence was confirmed by an IVIS Lumina system (Perkin Elmer, Waltham, MA, USA) equipped at Ewha Drug Development Research Core Center.

hEPO mRNA‐loaded LNPs were also administered via retro‐orbital injection at an mRNA dose of 0.1 mg/kg. For efficacy and immunogenicity comparisons between different ionizable lipids, the blood was collected after 6 h of injection via cheek bleeding and the serum was separated from the blood. hEPO and MCP‐1 levels were confirmed using ELISA kits. For time‐course confirmation of the hEPO protein level, LNPs were injected at an mRNA dose of 0.1 mg/kg. The serum was separated at 0 to 48 h after the injection and the protein level was quantified via an ELISA assay.

### 4.5 Preparation of single cells and antibody labeling for flow cytometry

To confirm tdTomato‐positive cells in the different cell types of the lungs, Cre mRNA was injected twice into mice at an mRNA dose of 0.3 mg/kg. After 2 days of the last injection, tdTomato fluorescence in five organs (heart, lungs, liver, spleen, and kidney) was confirmed using an IVIS.

For the preparation of lung single cells, the removed lung tissue was minced and incubated in 10 mL of digest medium at 37°C for 30 min. The digest medium was prepared by adding 10 mg of Liberase, 10 mg of hyaluronidase, and 50 units of DNase I to RPMI 1640. Digested lung tissue was transferred into a 70 μm nylon mesh strainer. The filtered cell suspension was centrifuged at 500×*g* for 5 min. The supernatant was then removed, and 10 mL of ammonium chloride solution was added to the cell pellet. After 10 min of incubation, 20 mL of RPMI 1640 solution were added to the solution and centrifuged at 500×*g* for 5 min. The supernatant was then removed, and 1 mL of cell staining buffer (BioLegend, San Diego, CA, USA) was added to the cell pellet. Next, the resuspended cells were labeled with antibodies. The antibodies used in this study were APC anti‐mouse CD31 (BioLegend; 102409), FITC anti‐mouse CD45.2 (BioLegend; 109805), and PE/Cyanine7 anti‐mouse CD326 (BioLegend; 118216). For staining of fibroblasts, APC anti‐mouse CD45.2 (BioLegend; 103111), APC anti‐mouse CD31 (BioLegend; 102409), APC anti‐mouse CD324 (BioLegend; 147311), PE/Cyanine7 anti‐mouse CD140a (BioLegend; 135911), Brilliant Violet 421™ anti‐mouse CD90.1 (BioLegend; 202529), and FITC anti‐mouse Sca‐1 (BioLegend; 122505) were used. Gating strategies for tdTomato‐positive expression in lung cells are described in Figure S13.

## AUTHOR CONTRIBUTIONS

**Minjeong Kim:** Conceptualization (equal); investigation (equal); writing – original draft (equal). **Michaela Jeong:** Conceptualization (equal); investigation (equal); writing – original draft (equal); writing – review and editing (lead). **Gyeongseok Lee:** Investigation (supporting). **Yeji Lee:** Investigation (supporting). **Jeongeun Park:** Investigation (supporting). **Hyein Jung:** Investigation (supporting). **Seongeun Im:** Investigation (supporting). **Joo‐Sung Yang:** Resources (equal). **Kyungjin Kim:** Resources (equal); supervision (supporting). **Hyukjin Lee:** Supervision (lead).

## CONFLICT OF INTEREST STATEMENT

The authors declare that there is no conflict of interests.

### PEER REVIEW

The peer review history for this article is available at https://www.webofscience.com/api/gateway/wos/peer-review/10.1002/btm2.10556.

## Supporting information


**Data S1:** Supporting information.

## Data Availability

The data supporting this study's findings are available from the corresponding authors upon reasonable request.
