# Supplementary material for: Novel piperazine‐based ionizable lipid nanoparticles allow the repeated dose of mRNA to fibrotic lungs with improved potency and safety
Source: Bioeng Transl Med. 2023 May 28;8(6):e10556. doi: 10.1002/btm2.10556 (PMC10658549; doi:10.1002/btm2.10556)
Supplement: Supplementary file 1 — Data S1: Supporting information. [file BTM2-8-e10556-s001.docx]

**Supporting information**

**Novel piperazine-based ionizable lipid nanoparticles allow the repeated dose of mRNA to fibrotic lungs with improved potency and safety**

Minjeong Kim^1,2+^, Michaela Jeong^1+^, Gyeongseok Lee^1^, Yeji Lee^1^, Jeongeun Park^1^, Hyein Jung^1^, Seongeun Im^1^, Joo-Sung Yang^3^, Kyungjin Kim^3^, Hyukjin Lee^1,^ *

^1^College of Pharmacy, Graduate School of Pharmaceutical Sciences, Ewha Womans University, Seoul 03760, South Korea

^2^Department of Biochemistry, Simmons Comprehensive Cancer Center, The University of Texas Southwestern Medical Center,

Dallas, TX 75390, USA

^3^ST Pharm Co., Ltd., Seoul 06170, South Korea

^+^ These authors contributed equally

*Corresponding author

Tel: +82-2-3277-3026 / Fax: +82-2-3277-2851

E-mail address: hyukjin@ewha.ac.kr (Hyukjin Lee)

**a**

**
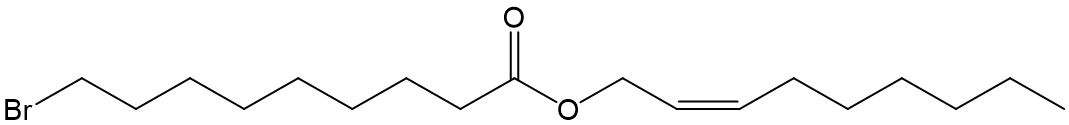
**

(Z)-non-2-en-1-yl 9-bromononanoate

**
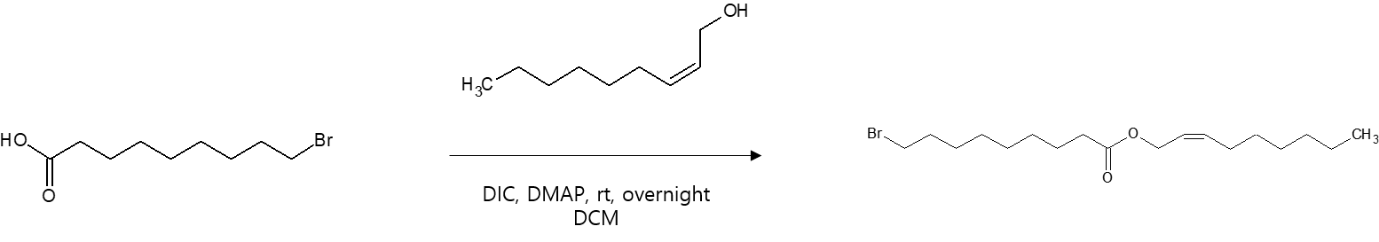
**

**Supplementary figure 1.** (a) Synthesis of (Z)-non-2-en-1-yl 9-bromononanoate. To a solution of 9-Bromononanoic Acid (1 g, 4.22 mmol) in DCM (10 mL) was added cis-2-Nonen-1-ol (0.4 g, 2.81 mmol), DIC (0.532 g, 4.22 mmol), and DMAP (0.0687 g, 0.562 mmol). The mixture was stirred for 16 hours at room temperature. The mixture was filtered and the filtrate was purified by column chromatography using a CombiFlash Rf system (0-10% Ethyl acetate in Hexane) to give (Z)-non-2-en-1-yl 9-bromononanoate (823 mg). m/z calcd for C18H33BrO2+ (M+H), 361.36; found, 361.36.

**
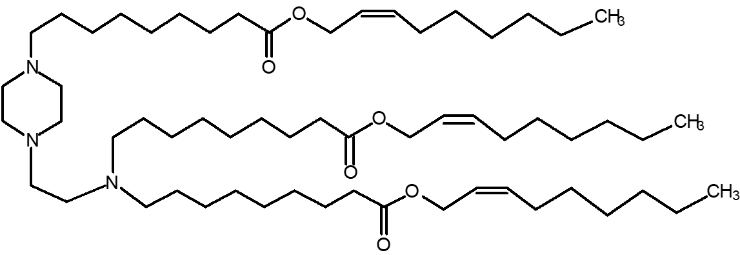
b**

244-cis


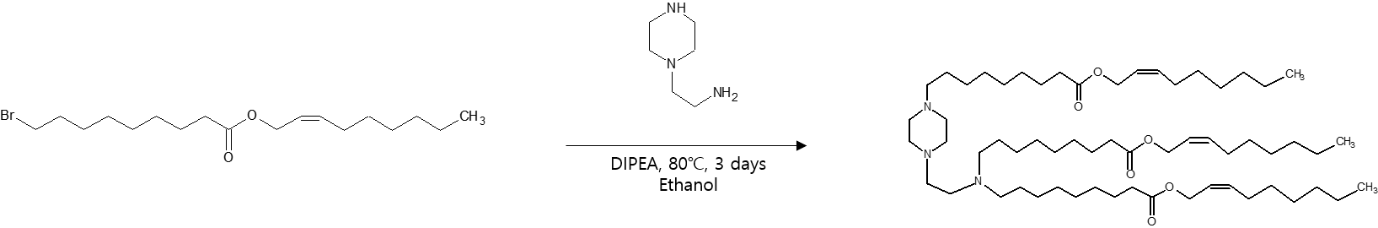


(b) Synthesis of 244-cis. To a solution of (Z)-non-2-en-1-yl 9-bromononanoate (0.8 g, 2.21 mmol) in Ethanol (10 mL) was added 1-(2-Aminoethyl)piperazine (0.092 g, 0.714 mmol), and DIEPA (0.086 g, 2.21 mmol). The mixture was stirred for 72 hours at 80 ℃. The mixture was cooled to room temperature and the solvent was removed. The resultant was purified by column chromatography using a CombiFlash Rf system (0-10% MeOH in DCM) to give 244cis (228 mg). m/z calcd for C60H112N3O6+ (M+H), 969.85; found, 970.8541.


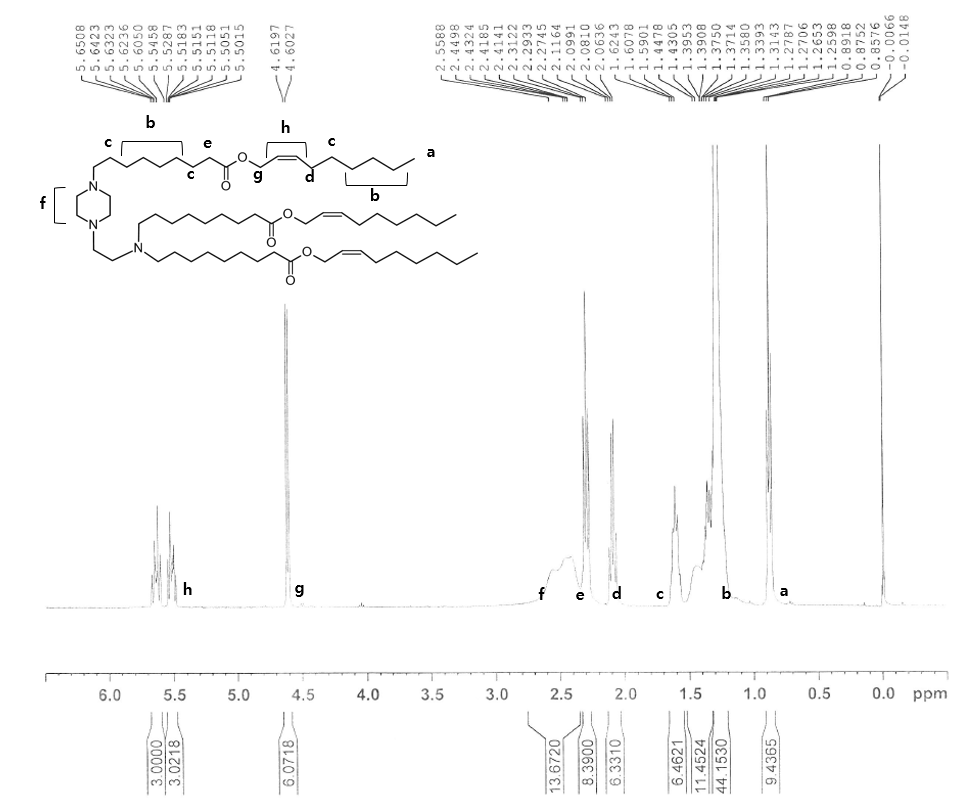


Supplementary figure 2. The NMR structure of 244cis ionizable lipid.


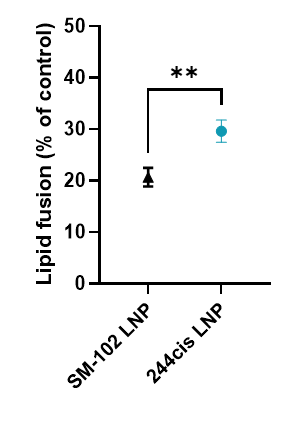


Supplementary figure 3. Lipid fusion of 244cis LNP and SM-102 LNP were determined by a FRET assay at pH 5.5.

a

|  | **Encapsulated efficiency (%)** | **Size**  **(nm)** | **PDI** | **Zeta potential**  **(mV)** |
| --- | --- | --- | --- | --- |
| C16-PEG2k LNP | 87.2 | 81.0 ± 5.5 | 0.09 ± 0.01 | -3.7 ± 0.16 |
| DMG-PEG2k LNP | 83.3 | 60.09 ± 5.1 | 0.16 ± 0.026 | -3.27 ± 0.32 |

b

**Supplementary figure 4.** The comparison of delivery efficacy of LNPs using two different PEG lipids (C16-PEG2k ceramide, DMG-PEG2k). (a) Physicochemical properties of both LNPs. (b) Evaluation of delivery efficacy by human erythropoietin(hEPO) expression. Both LNPs were systemically injected at 0.1 mg/kg of hEPO mRNA into Balb/c. After 6 hr injection, blood was collected and analyzed by ELISA (n=5).


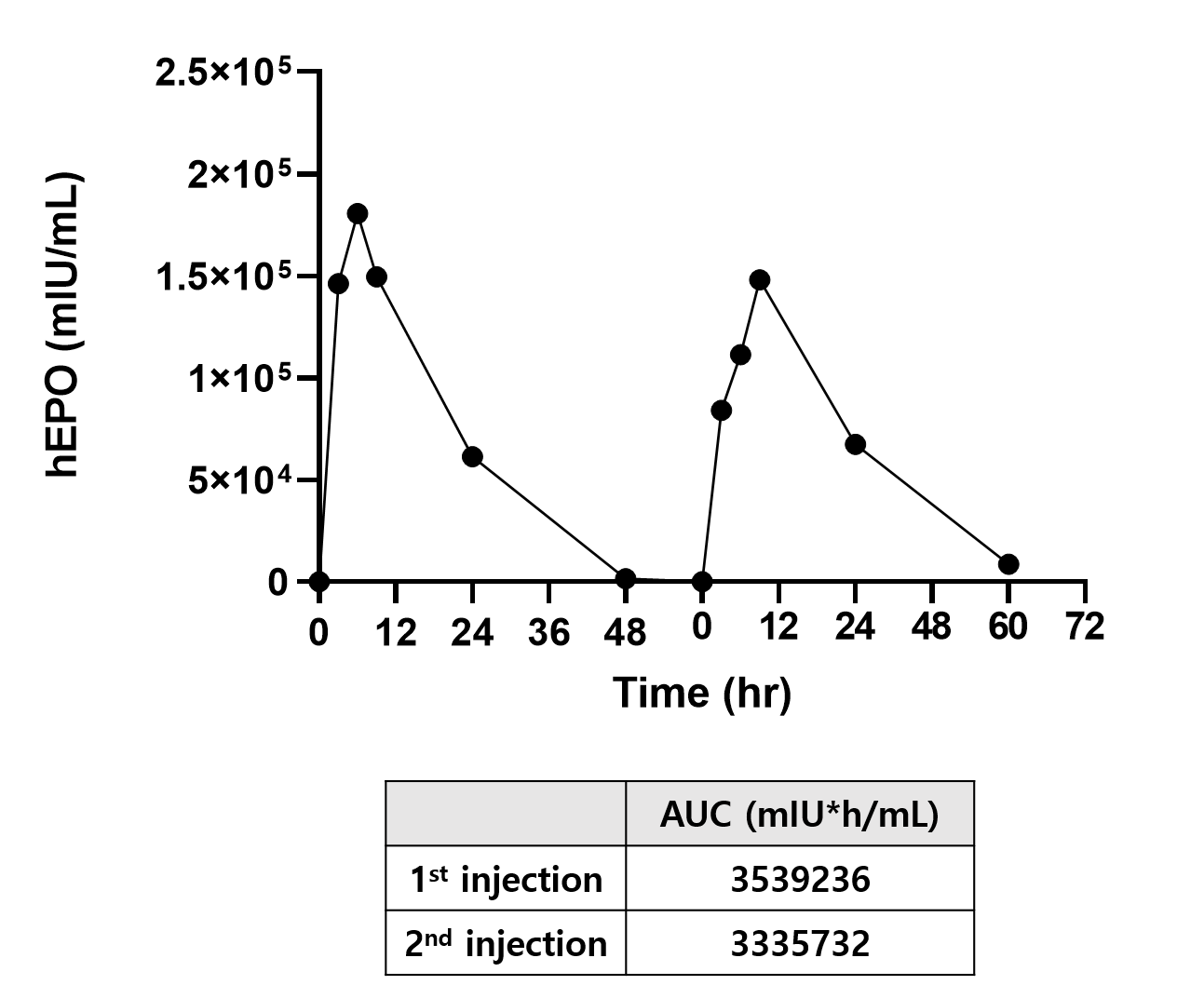
**Supplementary figure 5.** hEPO serum concentrations after the repeated administration of 244cis LNPs. ICR mice (n=4) were injected i.v. with hEPO mRNA formulated with LNP as intervals of 2 week. The blood was collected at 3, 6, 9, 24 and 48 hrs after injection and hEPO expression was measured by ELISA. AUC values of each injection were comparable.


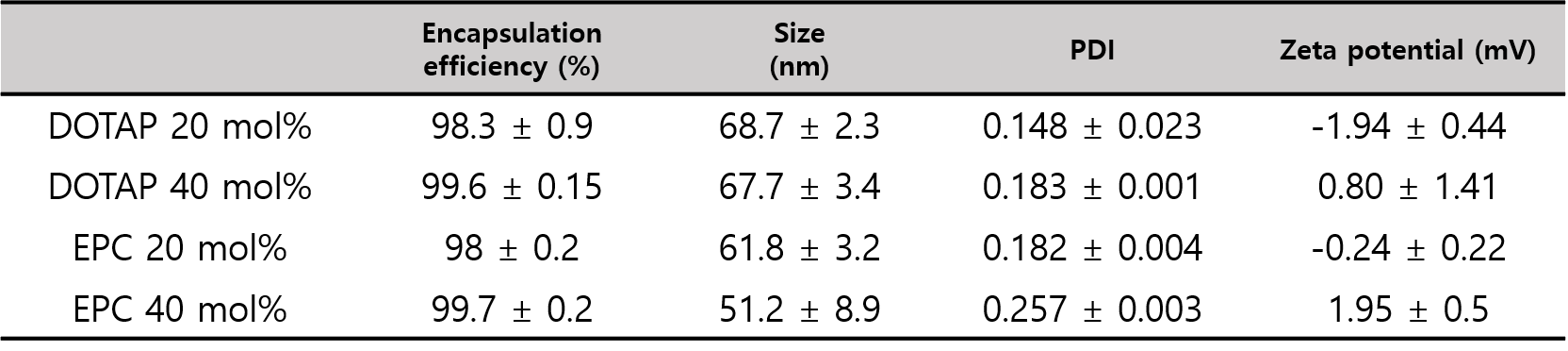


**Supplementary figure 6.** Physicochemical properties of the optimized LNPs; encapsulation efficiency (%), size (nm), PDI, and zeta potential (n = 3, mean ± SD).


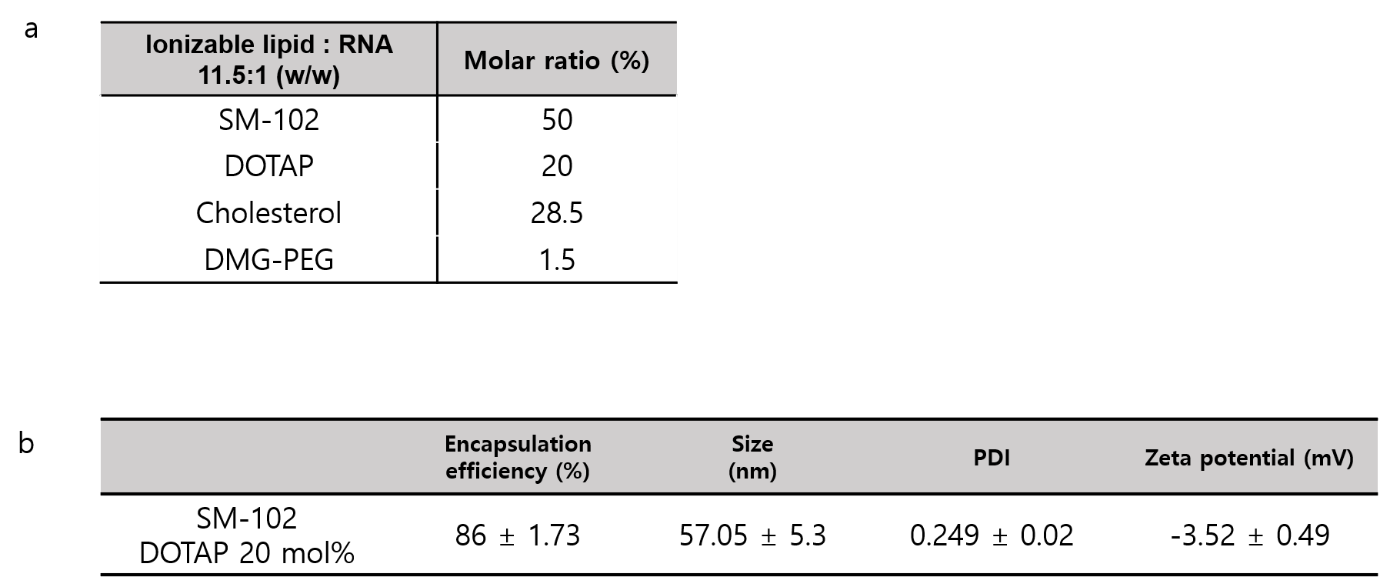


**Supplementary figure 7.** (a) The formulation ratio of SM-102 LNP with DOTAP and (b) physicochemical properties of SM-102 DOTAP LNPs; encapsulation efficiency (%), size (nm), PDI, and zeta potential (n = 3, mean ± SD).


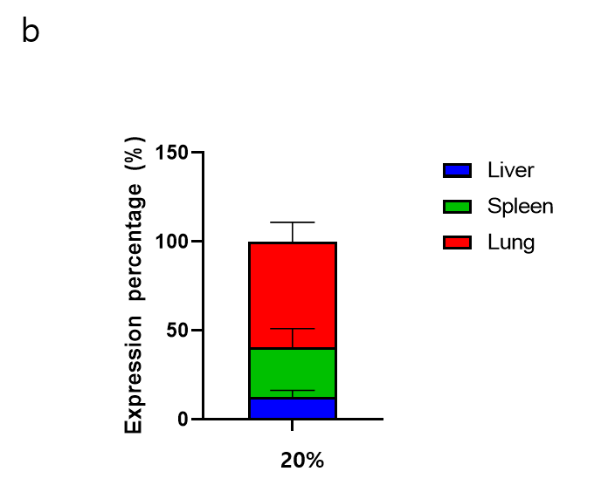

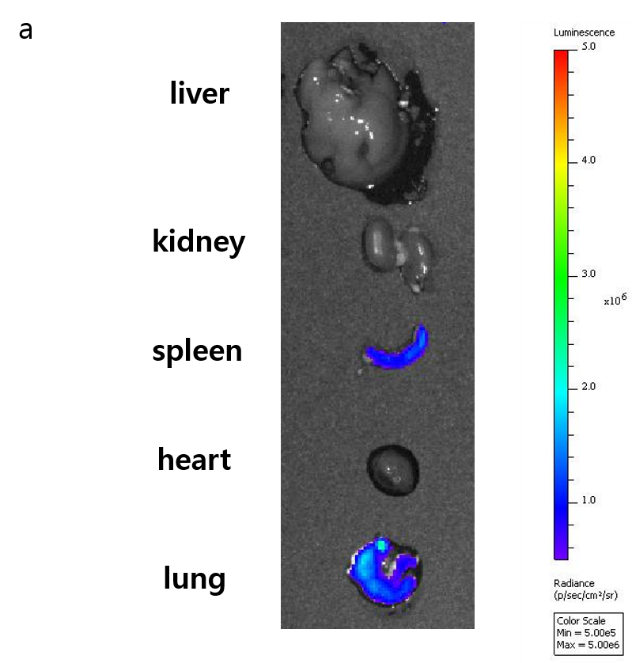


**Supplementary figure 8.** The biodistribution of SM-102 LNP incorporating 20% of DOTAP lipid. (a) Evaluation of delivery efficacy to the lungs by firefly luciferase expression. (b) The targeting efficiency of SM-102 LNP incorporating 20% of DOTAP lipid.


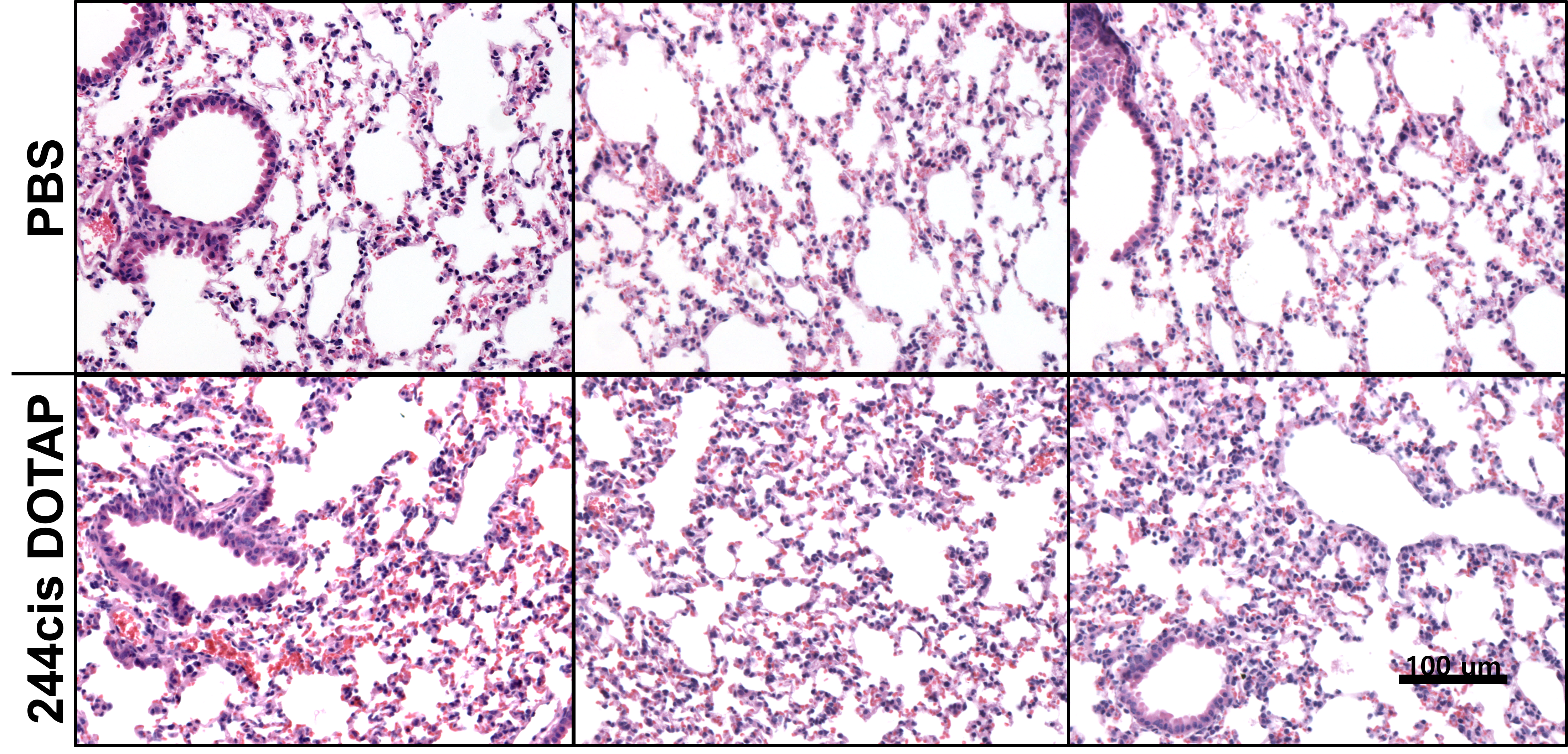


**Supplementary figure 9.** Histological evaluation of lung tissue. The H&E staining of PBS and 244cis DOTAP LNP treated lung section. The scale bars indicate 100 μm.

s


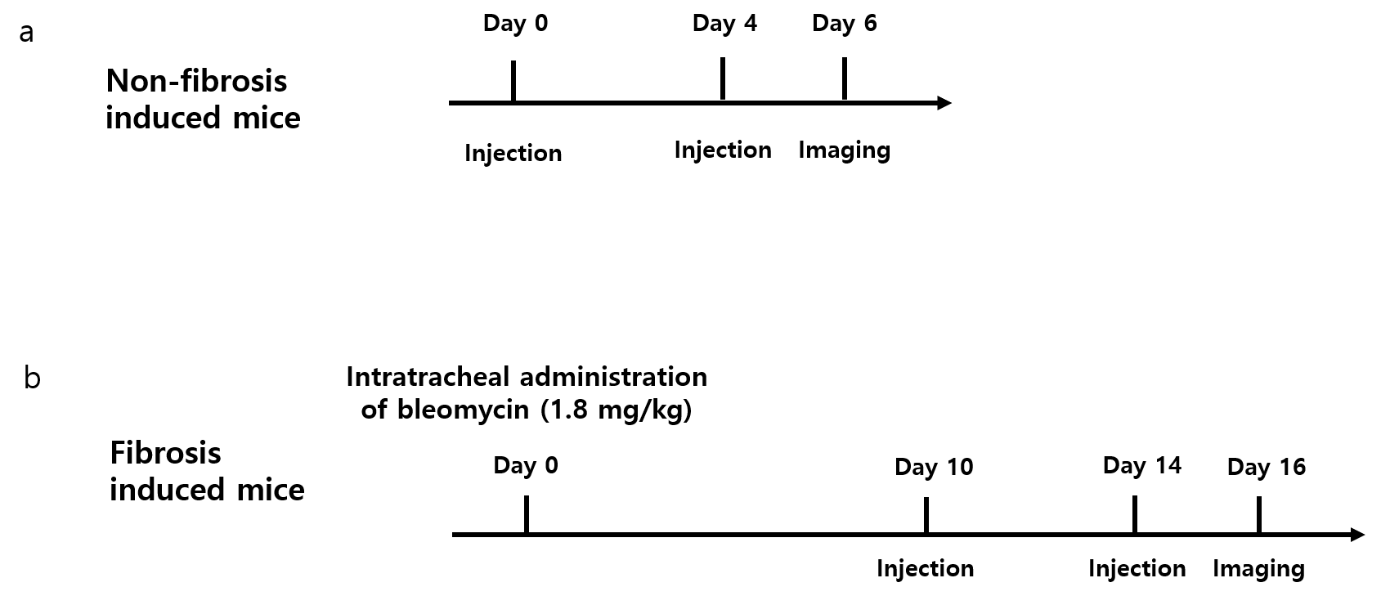


**Supplementary figure 10.** Experimental schedule of LNP administration in (a) non-PF model and (b) PF-induced model.

**
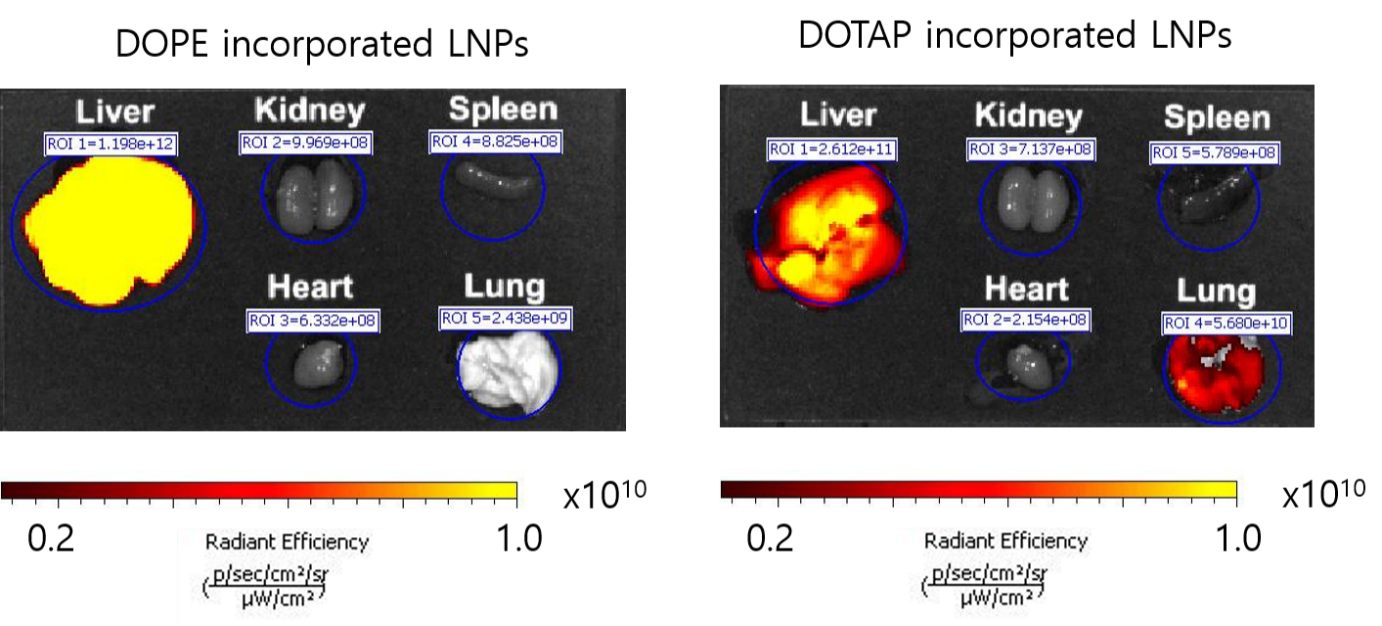
**

**Supplementary figure 11.** In vivo fluorescence imaging of 5 major organs extracted from LSL-tdTomato mice. tdTomato fluorescence were highly detected in the liver of mice with DOPE incorporated LNPs.


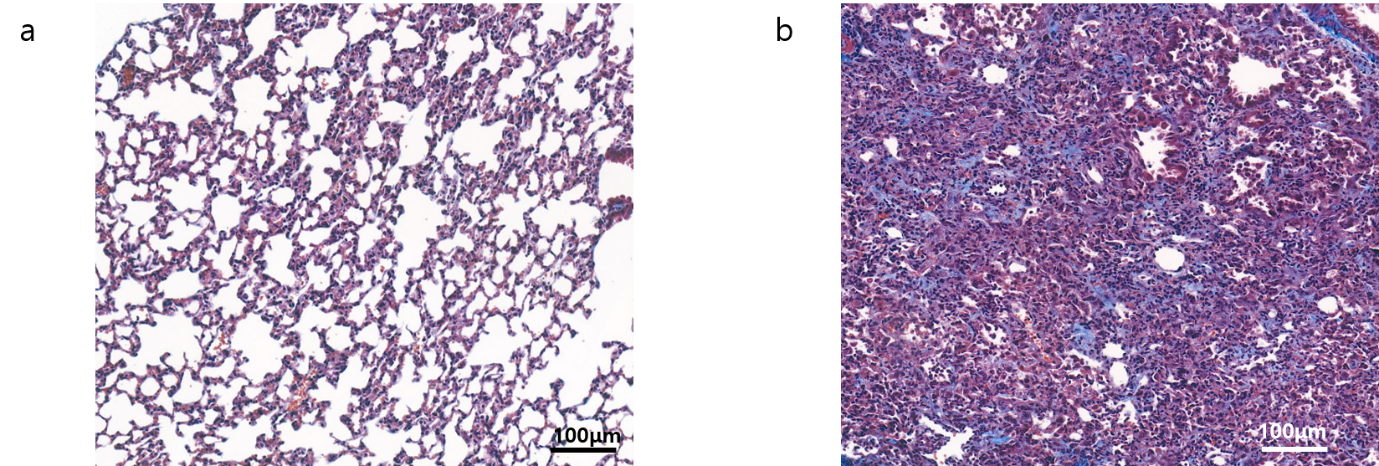


**Supplementary figure 12.** The Masson Trichrome staining of (a) non-PF lung and (b) PF-induced lung 14 days after BLM treatment. In the fibrotic regions, blue-colored collagen deposition and fibrotic were observed. The scale bars indicate 100 μm.

**Non-PF Lung**


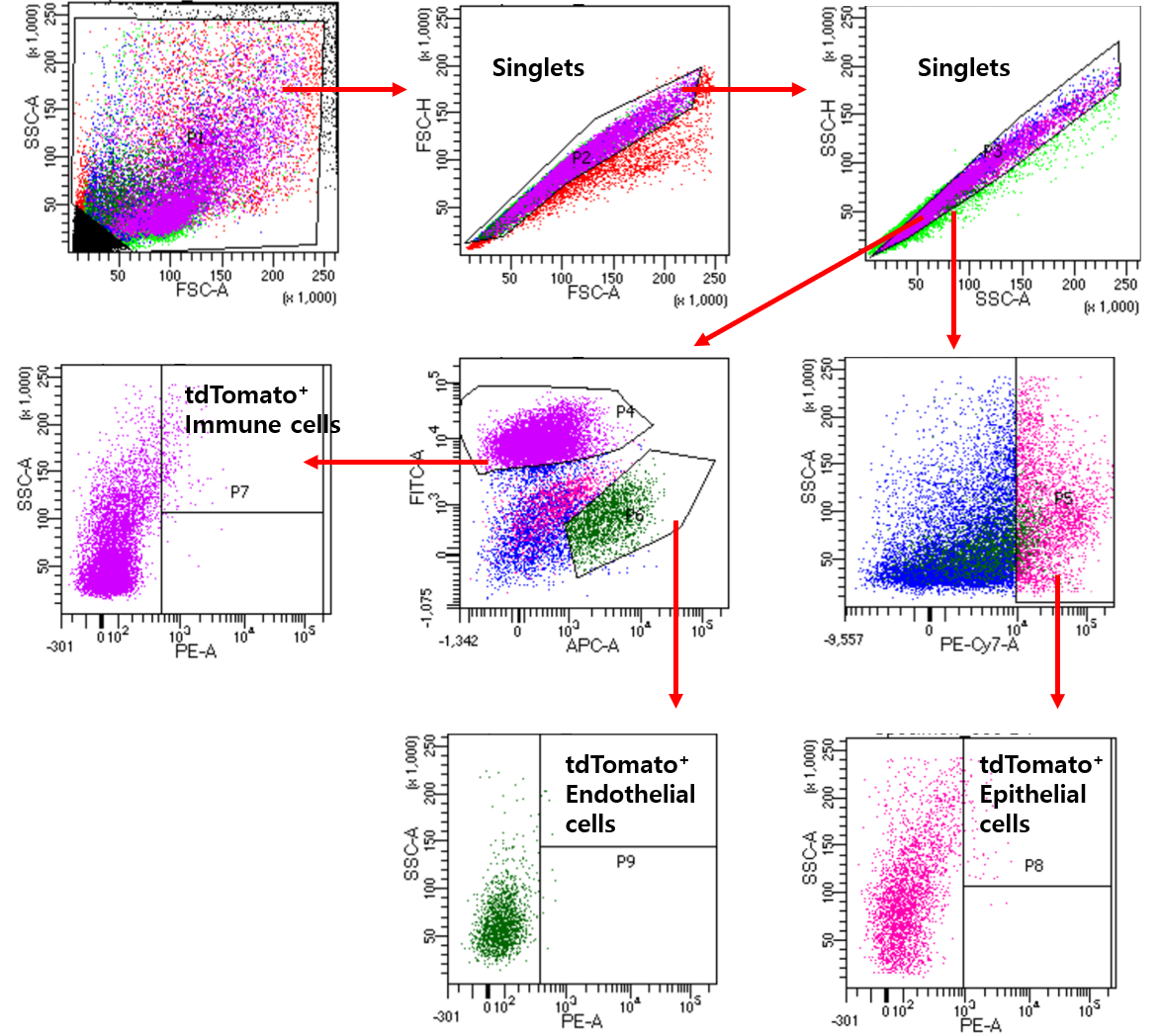
**a**

**PF-induced Lung**

**b**

**
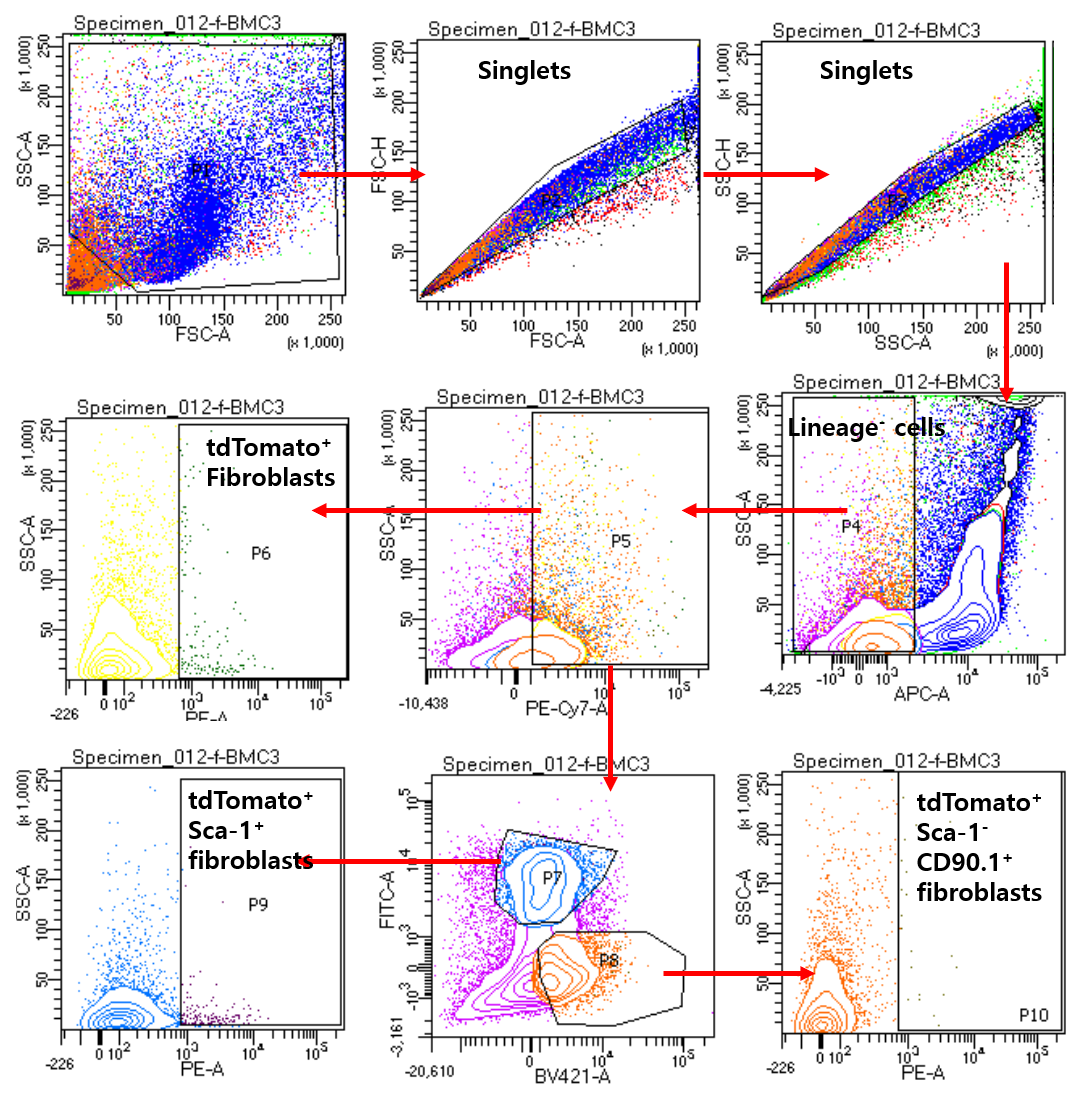
**

**Supplementary figure 13.** The flow cytometry gating strategy for analysis of tdTomato+ cells in (a) non-PF lung and (b) PF-induced lung. CD326+ was used to define epithelial cells, CD45+ and CD31- were used to define immune cells, and CD45-, CD31+ were used to define endothelial cells. For staining of fibroblasts, CD45.2-, CD31-, and CD324- were used to define Lineage cells and CD45.2-, CD31-, CD324-, and CD140a+ were used to defined fibroblasts. Sca-1+ of lineage cells were defined as Sca-1+ fibroblasts and Sca-1-, CD90.1+ of lineage cells were defined as Sca-1-, CD90.1+ fibroblasts. Mice were injected with 0.3 mg/kg at a dose of Cre mRNA. tdTomato positive cells was detected by flow cytometry after two days injection (n=3).
